# Negatively charged AuNP modified with monoclonal antibody against novel tumor antigen FAT1 for tumor targeting

**DOI:** 10.1186/s13046-015-0214-x

**Published:** 2015-09-15

**Authors:** Li Fan, Susanna Campagnoli, Hong Wu, Alberto Grandi, Matteo Parri, Elisa De Camilli, Guido Grandi, Giuseppe Viale, Piero Pileri, Renata Grifantini, Chaojun Song, Boquan Jin

**Affiliations:** Department of Pharmaceutical Chemistry and Analysis, School of Pharmacy, the Fourth Military Medical University, Xi’an, Shaanxi 710032 China; Externautics SpA, Siena, SI 53100 Italy; Department of Immunology, the Fourth Military Medical University, Xi’an, Shaanxi 710032 China

**Keywords:** FAT1, Negative gold nanoparticles, mAb198.3, Tumor targeting

## Abstract

**Background:**

Herein, we demonstrated the use of a newly generated anti FAT1 antibody (clone mAB198.3) for intracellular delivery of anionic gold NPs, to form active targeting Au nanoparticles with high payload characteristics.

**Methods:**

In vitro characterizations were determined by DLS, confocal microscopy, TEM, western blot, MALDI-TOF MS/MS analysis, MTT, ICP-MS and flow cytometry analysis. In vivo targeting efficacy was investigated by in vivo bio-imaging study and ICP-MS.

**Results:**

The specificity of the FAT1 recognition in colon cancer was confirmed by pre-adsorbing mAb198.3, adsorption dramatically abolished the antibody reactivity on colon cancer, thus confirming the binding specificity. The DLS size distribution profile of the AuCOOH, AuCOOH(Cy5)_ mAb198.3, AuCOOH(Cy5)_isotype has showed that the modified gold nanoparticles are well dispersed in water, PBS buffer and cell culture medium with 10 % FBS. By TEM measurement, the size of Au nanoparticles with spherical morphology is about 10–20 nm. AuCOOH_198.3 NPs were stable in an acidic environment, as well as in PBS buffer, cell culture media and media with 10 % serum. MTT results revealed that Au nanoparticles have well biocompatibility. TEM results indicated that conjugation of mAb198.3 on Au nanoparticles can be an effective delivery vehicle for negatively charged gold nanoparticles and increased its intracellular transport. It was also demonstrated by confocal microscopy that AuCOOH(Cy5)_mAb198.3 could attach to the cell membrane in very short time, then gradually delivered into cells. After 4 h incubation, almost all AuCOOH(Cy5)_mAb198.3 have been uptaken into or surrounding the cytoplasm and nucleus. In vivo results showed that only about 20 % of AuCOOH accumulated in tumor site due to EPR effect, while nearly 90 % of AuCOOH_mAb198.3 was found in tumor, providing sufficient evidence for receptor-specific targeting by mAb198.3.

**Conclusion:**

According to in vitro and in vivo research results, the intracellular uptake of negatively charged AuCOOH_mAB198.3 particles is enhanced to a greater extent. Thus, AuCOOH_mAb198.3 holds significant potential to improve the treatment of cancer.

**Electronic supplementary material:**

The online version of this article (doi:10.1186/s13046-015-0214-x) contains supplementary material, which is available to authorized users.

Nanoscaled drug carriers have been used widely for drug delivery such as liposomes [1, 2], microspheres [3–6], polymeric shells [7], micelles, niosomes, nanoparticles [8], dendrimers and nanofibers [9]. The drug was loaded on or into these nanoscaled materials by several different techniques, such as encapsulation, surface loading, hydrogen bonding, and other types of interactions. However, the drug loading efficiency of the current nanoparticle drug delivery systems based on polymers are still low [10]. Therefore, improving loading efficiency is a critical factor in the design of drug delivery systems. Au nanoparticles can offer significant advantages over these delivery mechanisms in terms of high stability [11], high specificity [12], high drug carrying capacity [13], ability for controlled release [14] and the capability to transport both hydrophilic and hydrophobic molecules.

The presence of phospholipids on the mammalian cell membrane imparts a net negative charge [15], restricting anionic entities to bind and subsequent transport into the cell. For example, Xia et al. have demonstrated that the membrane affinity constant as well as the rate of internalization of cationic NPs is significantly higher than that of anionic NPs in human cancer cells [16]. Despite high uptake efficiency, cationic NPs tend to be toxic [17] and can elicit immunotoxic [18] and genotoxic [19] responses in a variety of cells. In contrast, anionic nanoparticles are nontoxic [20] and minimize the protein adsorption on their surfaces [21], thereby improving the pharmacokinetic profile [22]. Using an *in vitro* tumor model, we have also shown that the anionic gold NPs can diffuse faster and would be a better candidate to deliver drugs deep inside the tissues [23]. Therefore, strategies to enhance the intracellular uptake of negatively charged NPs can aid the drug penetration into the tumor core, circumventing the possible cytotoxicity issues.

FAT1 is a surface exposed protein. It belongs to the human FAT gene family, a subclass of the cadherin superfamily composed of four giant proteins (from FAT1 to FAT4) of 500–600 kDa sharing structural similarities from invertebrates to mammals. Human FAT1 is a typeI transmembrane protein composed of 34 cadherin repeats, five EGF-like repeats, a laminin A-G domain in the extracellular region and a cytoplasmic tail that is quite distinct from classical cadherins [24, 25]. The protein was recently identified as a novel colorectal cancer (CRC)-associated marker (Grifantini et. al., submitted manuscript) by an immune-histochemical screening of a collection of antibodies towards membrane-associated and secreted proteins so far marginally characterized in the scientific literature [24]. One particular mAb was generated in our lab named as mAb198.3. MAb198.3 recognizes the FAT1 protein in CRC, in which is gives a predominant membranous staining (Grifantini et. al., submitted manuscript). Moreover, mAb198.3 is rapidly internalized when it binds to FAT1-expressing colon cell lines (Grifantini et. al., submitted manuscript). Based on these findings, this novel marker and specific mAb could offer new opportunities for negative gold nanoparticles intracellular delivery, cancer diagnosis and treatment [26].

In this study, by using an independent collection of clinical samples, we further confirmed that mAb198.3 recognizes FAT1 in 79 % colon adenocarcinomas, while it is negative or marginal expressed in most normal human tissues when tested on 24 different healthy human tissues analyzed by immunohistochemistry (IHC). Moreover, we investigated the internalization properties of mAb198.3 bound to nanoparticles. MAb198.3 was designed to conjugate on Au nanoparticles backbone to form active targeting Au nanoparticles with high payload characteristics. Herein, we demonstrated the use of mAb198.3 for intracellular delivery of anionic gold NPs (Fig. 1). Results showed that conjugation of mAb198.3 on anionic gold nanoparticles could effectively deliver particles into tumor cells or tissues but rarely into normal cells. This active targeted delivery system with high payload capacity could be considered as a promising targeting anti-tumor drug delivery system.

**Fig. 1:**
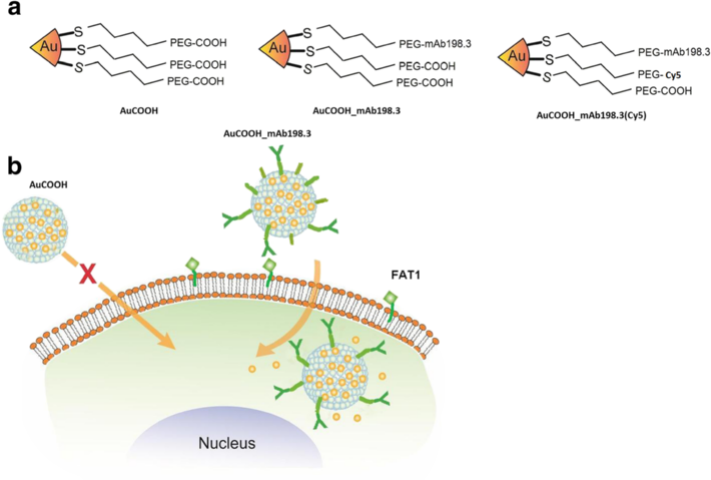
Intracellular negative gold nanoparticles delivery. **a** Configurations of Au nanoparticles. **b** Schematic of FAT1 mediated endocytosis and drug release of AuCOOH_mAb198.3

## Experimental section

### Materials

All reagents were purchased from Sigma Aldrich without purification, unless otherwise mentioned. Dichloromethane (DCM) as a solvent for chemical synthesis was dried to remove excess water. NMR spectra were detected by a Bruker AVANCE 400 machine. TEM images were acquired on a JEOL 7C at 80 keV. Dynamic light scattering (DLS) data were measured with a Malvern Zetasizer Nano ZS.

### Methods

#### Cloning-expression-purification of recombinant FAT1

The FAT1 domain spanning from amino acid 723 to amino acid 1352 of the protein was selected for cloning and expression in *E.coli*. For cloning, cDNA pools were generated by reverse transcription from total RNAs (Euroclone) of human tissues (bone marrow, fetal brain, placenta and testis) and pooled. The corresponding cDNA fragment was amplified using specific primers (selected using Primer 3 Software, Premier Biosoft International) so as to fuse a 10 histidine tag sequence (MGSDKIHHHHHHHHHHGV) at the 5′ end, annealed to in-house-developed vector (p2N) derivative of vector pSP73 (Promega) adapted for the T4 ligation independent cloning method [27]and used to transform *E.coli* NovaBlue cell recipient strain*. E. coli* transformants were plated onto selective LB plates containing 100 μg/ml ampicillin (LB Amp), and positive *E.coli* clones were identified by restriction enzyme analysis of purified plasmid followed by DNA sequence analysis. For expression, the p2N-FAT1 plasmid was used to transform BL21-(DE3) *E.coli* cells. Recombinant FAT1 (rFAT1) was purified from the inclusion bodies. Briefly, *E.coli* BL21-(DE3) cells harboring the p2N-FAT1 were inoculated in 50 ml of ZYP-5052 growth medium and grown at 37 °C for 24 h. Afterwards, bacteria were harvested by centrifugation and lysed into 10 ml of B-Per Reagent (Pierce) containing 1 mM MgCl_2_, 10 units/ml of DNAse I (Sigma), and 1 mg/ml Lysozyme (Sigma). After 30 min at room temperature under gentle shaking, the insoluble inclusion bodies were collected by centrifugation at 30.000 × g for 40 min at 4 °C. To solubilize inclusion bodies, the pellet was resuspended in 10 ml of 6 M guanidine hydrochloride, 1 mM Tris(2-carboxyethyl)-phosphine hydrochloride (TCEP) (Pierce), and 40 mM Tris–HCl pH 8. After clarification by centrifugation at 30.000 g for 30 min, the supernatant was loaded on 0.5 ml columns of Ni-activated Chelating Sepharose Fast Flow resin (GE-Healthcare). After washing the resin with 10 column volumes of 6 M urea, 60 mM imidazole, 0.5 M NaCl, 1 mM TCEP, and 50 mM Tris–HCl pH 8, recombinant proteins were eluted with the same buffer containing 500 mM imidazole. The purity of rFAT1 was analyzed by SDS-Page and its concentration was determined by Bradford assay using the BIORAD reagent (BIORAD) with a bovine serum albumin standard according to the manufacturer’s recommendations. Moreover, the identity of the protein was verified by MALDI-TOF MS/MS analysis as described [24].

#### Production of hybridomas and purification of mAbs

Hybridoma cells were generated according to our standard protocol [28]. BALB/c mice (8 weeks old, female) were immunized with 20 μg of rFAT1, mixing with complete Freund’s adjuvant by subcutaneous (s.c.) injection and immunized every 4 weeks using 20 μg of rFAT1. 10 days after final immunization, the concentration of anti-FAT1 antibodies in sera was monitored by indirect ELISA. Splenocytes and SP2/0 myeloma cells were mixed in 50 % PEG solution (Mw4000). The positive hybridomas were selected and subcloned.

BALB/c mice were inoculated with 1 × 10^6^ hybridoma cells by i.p. injection and the ascitic fluid was collected after 7–14 days. The fraction of mAbs in ascites was precipitated with 50 % ammonium sulphate and futher purified by ProteinG-Sepharose CL-4B chromatography. Obtained IgG fractions were dialysis in PBS and stored at −70 °C.

#### Cell culture

Colo 205 cells (Human Colon Carcinoma) and HeLa cells were cultured in RPMI 1640 and DMEM, respectively, supplemented with 10 % fetal bovine serum and 1 % antibiotics. The cells were maintained at 37 °C incubator with 5 % CO_2_.

#### Cell transfection

For transfection experiments, the FAT1 encoding plasmid was generated by PCR amplifying the full length coding region specific primer pairs (acid 723 to amino acid 1352, Forward: 5′-gaattcggtattcaggtaaaggaaaacc-3′; Reverse: 5′-ctcgagctaggacggtttgggcttggag-3′), and the PCR products were cloned into plasmid pcDNA3 (Invitrogen) and sequence-verified. HeLa cells (400,000 per well, in 6-well plates) were transiently transfected with 4 micrograms of the resulting plasmids and with the empty vector as negative control (mock) using the Lipofectamine-2000 transfection reagent (Invitrogen). Cells were collected for Western blot analysis 24 h after transfection and analysed for FAT1 expression by Western blot.

#### Western blot

Sub-confluent cell mono-layers were detached with PBS-0.5 mM EDTA and lysed by several freeze-thaw passages in PBS-1 % Triton. Total protein extracts were loaded onto a SDS-Page (2x10^5^ cells/lane) and expression of target proteins was assessed by Western blot analysis using the FAT1 specific mAb198.3. Western blot was performed by separation of the protein extracts on pre-cast SDS-PAGE gradient gels (NuPage 4-12 % Bis-Tris gel, Invitrogen) under reducing conditions, followed by electro-transfer to nitrocellulose membranes (Invitrogen) according to the manufacturer’s recommendations. The membranes were blocked in blocking buffer composed of 1x PBS-0.1 % Tween 20 (PBST) added with 10 % dry milk, for 1 h at room temperature, incubated with the antibody diluted 1:2500 in blocking buffer containing 1 % dry milk and washed in PBST-1 %. The secondary HRP-conjugated antibody (goat anti-mouse immunoglobulin/HRP, Perkin Elmer) was diluted 1:5000 in blocking buffer, and chemiluminescence detection was carried out using a Chemidoc-IT UVP CCD camera (UVP) and the Western Lightning™ Cheminulescence Reagent Plus (Perkin Elmer), according to the manufacturer’s protocol. Where indicated, antibodies used for Western blot were pre-adsorbed at 4 °C on membranes saturated with their respective recombinant proteins.

#### Confocal microscopy analysis of FAT1 expression

For confocal microscopy analysis of FAT1 expression, cells were seeded on cover slips. After 48 h incubation, cells were washed by PBS and fixed with 3 % formaldehyde solution for 20 min. After washing by PBS for several times, cells were permeabilized with 0.01 % BriJ96® (Fluka) and incubated with antibodies (1:200) overnight at 4 °C. Cells were then stained with Alexafluor 488-labeled goat anti-mouse antibodies (Molecular Probes). DAPI (Molecular Probes) was used to stain nuclei. The cells were mounted with glycerol plastine and observed using confocal microscope (LeicaSP5).

#### IHC staining

Tissue arrays of 24 normal human tissues (FDA808c) and colon carcinoma tissues (BC05118a) were purchased from US Biomax Inc. Tissue arrays were treated by dimethyl benzene and rehydrated using alcohols with different ratios. Methanol containing 3 % H_2_O_2_ was used for blocking endogenous peroxidase activity for 10 min, while nonspecific binding was blocked by PBS containing 10 % normal goat serum for 30 min. The slices were incubated with mAb198.3 (0.2 mg/L) for 1 h. Normal mouse IgG was collected as negative control. After washing by PBS for 3 times, the slices were dipped into biotin-conjugated goat anti-mouse IgG (Promega, Madison, USA) for 1 h at room temperature, incubated for another hour with streptavidin-HRP complex (Promega, Madison, USA). Then antibody complexes were stained by DAB chromogen. Tissue arrays were counterstained with Mayer’s hematoxylin for 30 s, followed by dehydrated through gradient ethanol, cleared in dimethyl benzene, mounted, and examined using light microscopy.

#### Preparation of poly(ethylene glycol) 2-mercaptoethyl ether acetic acid (COOH-PEG-SH) capped gold nanoparticles (AuCOOH)

2 nm gold NPs was synthesized following Brust- Schiffrin method [29] with some modification. An aqueous solution of hydrogen tetrachloroaurate (1 g, 250 ml) was mixed with a solution of tetraoctylammonium bromide in toluene (2.1 g, 500 ml). The two-phase mixture was vigorously stirred until all the tetrachloroaurate was transferred into the organic layer and C_5_SH (0.337 ml) was then added to the organic phase. A freshly prepared aqueous solution of sodium borohydride (2 g, use small amount of water to dissolve) was quickly added with vigorous stirring. After further stirring overnight the organic phase was separated, evaporated with rotary evaporator and mixed with 400 ml ethanol to remove excess thiol. The mixture was kept at −18^。^C and the dark brown precipitate was filtered off and washed with ethanol many times. The mole ratio of HAuCl_4_: TOAB: C_5_SH: NaBH_4_ = 1:1.5:1.2: 20.

COOH-PEG-SH capped gold nanoparticles (AuCOOH) were prepared from pentane thiol-capped gold NPs (~2 nm core) via a place exchange reaction. Briefly, 20 mg of pentane thiol-capped 2 nm gold NPs prepared from Brust-Schiffrin method [30] and 80 mg of COOH-PEG-SH were weighed in two separate vials and 5 ml dry DCMwas added to each of the vials. Under nitrogen atmosphere, COOH-PEG-SH solution was added dropwise to the gold NPs solution and stirred for 2 days. The black precipitation of AuCOOH was further washed with Hexane/DCM twice to remove free ligands, dried under reduced pressure and solubilized in distilled water. After 2 days of dialysis, the NPs were lyophilized and redissolved in MilliQ water.

#### Preparation of Cy5 labeled AuCOOH (AuCOOH_Cy5)

Cy5 as a fluorescence indicator was chosen to simulate antitumor drug and labeled in AuCOOH. Cy5 NHS ester was first reacted with cysteamine(SH-C_2_H_4_-NH_2_) to obtain SH-C_2_H_4_-NH-CO-Cy5. And SH-C_2_H_4_-NH-CO-Cy5 capped gold nanoparticles (AuCOOH_Cy5) were prepared via a place exchange reaction [31]. Briefly, 15 mg Cy5 NHS ester dissolved in 200 μL DMSO was dropwised added in cysteamine solution (1.89 mg, 3 mL 0.1 M PBS with pH 8.3–8.5) and stirred for 24 h at room temperature under dark. After reaction, the product SH-C_2_H_4_-NH-CO-Cy5 was lyophilized and redissolved in small amount of DMSO and then added to dichloromethane (DCM) for future use.

To obtain AuCOOH_Cy5, 6 mg of SH-C_2_H_4_-NH-CO-Cy5, 34 mg of COOH-PEG-SH and 10 mg of pentane thiol-capped 2 nm gold NPs were weighed in three separate vials and 2 mL dry DCM was added to each of the vials. Under nitrogen atmosphere, COOH-PEG-SH solution and SH-C_2_H_4_-NH-CO-Cy5 solution were added dropwise to the gold NPs solution and stirred for 2 days. The dark blue product of AuCOOH_Cy5 was further washed with Hexane/DCM twice to remove free ligands, dried under reduced pressure and solubilized in distilled water. After 2 days of dialysis, the NPs were lyophilized and redissolved in MilliQ water.

#### Preparation of mAb198.3 and isotype decorated AuCOOH_Cy5 (AuCOOH(Cy5)_ mAb198.3)

Attachment of mAb198.3 to the AuCOOH_Cy5 was performed using standard N-hydroxysuccinimide/1-ethyl-3-(3-dimethylaminopropyl) carbodiimide (NHS/EDC) coupling chemistry. Reaction raw molar ratio was as follow, AuCOOH_Cy5: EDC: NHS: mAb198.3 (1:10:10:5). Briefly, the carboxy groups on the particle surface are first activated by reaction with EDC hydrochloride and NHS in 0.1 M MES buffer (pH 5) for 1 h. Then mAb198.3 attachment was performed by adding the concentrated activated nanoparticles to a dilute PBS solution containing mAb198.3 at pH 7.4. The solution was mixed well and transferred into an Eppendorf tube and shaken gently overnight at 4 °C under dark. After reaction, free antibody and AuCOOH(Cy5)_mAb198.3 was separated by fast protein liquid chromatography (FPLC) to remove free antibody, NHS and EDC. The mobile phase was MilliQ water at a flow rate of 5 mL/min. The final molar ratio of AuCOOH_Cy5: mAb198 in AuCOOH(Cy5)_mAb198.3 was 1:4.

Attachment of isotype control to the AuCOOH_Cy5 was performed using the same protocol as AuCOOH(Cy5)_ mAb198.3 preparation by changing mAb198.3 to isotype control.

#### Characterization of Au nanoparticles

AuCOOH, AuCOOH_Cy5, AuCOOH(Cy5)_ mAb198.3 and AuCOOH(Cy5)_ isotype were analyzed by dynamic light scattering, NMR and TEM. Dynamic light scattering was performed with a Malvern Zetasizer Nano ZS to determine particles size and potential. Transmission electron microscopy was done to intuitive study the dispersibility and uniformity of particle on a JEOL 7C device at 80 kV. Samples were prepared by dilution of the nanoparticles in water, placement of a drop (2-3 μL) on a copper grid carrying a 20 nm thick carbon film (CF-300-Cu, Electron Microscopy Sciences), and drying for 1 h.

#### Evaluation of AuNP cytotoxicity on cell viability and proliferation

Colo 205 cells were seeded in a 96-well plate at a density ~15,000 cells/well 24 h prior to the experiment. Next day, the old media was replaced by different concentrations of AuCOOH in serum containing media, and the cells were further incubated for 24 h at 37 °C in a humidified atmosphere of 5 % CO_2_. The cells were washed with PBS three times and 10 % alamar blue in serum containing media was added to each well and further incubated at 37 °C for 4 h. The cell viability was then determined by measuring the fluorescence intensity at 590 nm using a SpectraMax M5 microplate spectrophotometer. Viability (%) of NP-treated cells was calculated taking untreated cells as 100 % viable. Each experiment was done in triplicate.

#### Flow cytometry assay

Reaction of the mAb198.3_Cy5, isotype antibody_Cy5, AuCOOH(Cy5)_ mAb198.3 and AuCOOH(Cy5)_ isotype with Colo205 cells was analyzed using flow cytometry. Colo 205 cells were harvested and incubated with free mAb198.3_Cy5, isotype antibody_Cy5, AuCOOH(Cy5)_ mAb198.3 and AuCOOH(Cy5)_ isotype (20nM) for 30 min at 4 °C. After incubation, cells were washed for three times and resuspended in PBS. Samples were analyzed using a FACSCalibur flow cytometer and CellQuest™ Pro software (BD Bioscience, San Jose, CA). Each experiment was done in triplicate.

#### Cell TEM analysis

It is important to know the fate of the nanoparticles after the treatment. Do the particles internalized by cells due to mAb198.3 on the particle surface or do the particles remain bound to the cell membrane? To address these issues transmission electron microscopy study following standard protocol was performed. Colo 205 cells were first treated with AuCOOH, AuCOOH(Cy5)_ mAb198.3 and AuCOOH(Cy5)_ isotype for 4 h, separately. After the incubation, cells were centrifuged gently at 1000 rpm for 5 min. The resultant cell pellets were then washed with PBS, and fixed in 2.5 % (v/v) glutaraldehyde in 0.1 M phosphate buffer, pH 7.2. All samples were then washed with PBS, pH 7.2, followed by a 1 h postfix in phosphate-buffered 1 % Osmium tetroxide (OsO_4_). After washing in distilled water, the cells were stained with 2 % uranyl acetate for 30 min at 60 °C. After washed for another three times, cells were dehydrated in progressively higher concentrations of ethanol and 100 % propylene oxide, and embedded in resin. Cells were cut into thin pieces on a Reichert Ultracut E ultramicrotome, placed on 200 mesh copper grids, and stained with lead citrate. Micrographs were taken on a JEOL 7C operating at 80 KV.

#### Antibody internalization

To demonstrate mAb198.3 mediated cell-selective intracellular delivery of the AuCOOH, cultured cells (5 × 10^5^/sample) were treated with the AuCOOH(Cy5)_ mAb198.3 and AuCOOH(Cy5)_ isotype for 12 h at 37 °C. Cells were then washed twice with PBS and fixed with 4 % triformol solution. Then the cells were stained with 1 μg/ml 4′–6-diamidino-2-phenylindole (DAPI, Invitrogen) for 8 min to label nuclei and washed with PBS for three times to remove free dye. Lastly, cell suspensions were added onto slide and examined using a laser scanning confocal microscope (Olympus, Fluo ViewTM 1000) at 400 × magnification.

#### Animal model

All animal studies were conducted under a protocol approved by National Institute of Biological Science and Animal Care Research Advisory Committee of Fourth Military Medical University. All experiments involving mice conducted following the guidelines of the Animal Research Ethics Board of Fourth Military Medical University. Six-week-old BALB/c athymic nude mice were purchased from Peking University Health Science Center and tumors were established by subcutaneously injecting Colo-205 cells of 0.10 mL volume containing 5 × 10^7^ cells/mL media into the right flank of the mice. The tumor sizes were monitored every other day and the animals were subjected to in vivo experiments when the diameter of the tumors reached 6 ~ 8 mm (typically 3–4 weeks after inoculation).

#### In vivo bio-imaging study

For in vivo bio-imaging, xenograft tumor mice received a tail intravenous injection of 2 μmol/L AuCOOH(Cy5)_ mAb198.3, AuCOOH(Cy5)_ isotype solution prepared in PBS (0.25 mL) and injection concentration of mAb198.3_Cy5, isotype antibody_Cy5 was 10 μmol/L (0.25 mL) due to conjugation ratio between Au and mAb was 1 to 5. The animals were fully anesthetized by inhalation of a mixture of oxygen with 5 % isoflurane 10–15 min 24 h post-injection. The in vivo bio-images were acquired using an in vivo fluorescence imaging system (Cambridge Research & Instrumentation, Inc., Maestro EX) with Ex = 640 nm.

Tissues were harvested from the mice after in vivo bio-imaging studies to quantitative analyze nonspecific accumulation of Cy5 in other organs besides tumor. The mice were sacrificed by cervical dislocation. Heart, liver, spleen, lung, kidneys and tumor, along with peripheral blood in the chest were collected. Organs were washed by PBS buffer twice and images were also acquired using the fluorescence imaging system with Ex = 640 nm.

#### ICP- MS measurements

Inductively coupled plasma mass spectrometry (ICP- MS) could detect element type and concentration in the samples. Quantification of gold nanoparticle uptake by cells is best performed by this technique that has high specificity and low limits of detection. ICP- MS has excellent limits of detection (18 parts per trillion for gold) and can be applied to quantify the cellular uptake by digesting the cells with strong acid. The amounts of Au uptake were measured on a Perkin-Elmer Elan 6100 mass spectrometer. Each sample was measured in triplicate. Colo-205 cells were seeded in 24 well plates at 30,000 cells in 0.5 mL medium 24 h prior to the experiment. Next day, the cells were washed with PBS and incubated with AuCOOH, AuCOOH_isotype and AuCOOH_mAb198.3 NPs (150nM Gold nanoparticles) for 6 h cells were washed three times with PBS and lysed. Cell lysate was digested with 0.5 mL aqua regia for 4 h. After digestion, each sample was diluted into 10 mL de-ionized water, and subjected to ICP-MS analysis. Au uptake in vivo by tumor was also investigated by ICP-MS following the same method.

## Results and discussion

### Generation and characterization of mAbs against FAT1

Our approach to discover novel potential markers and therapeutic targets is based on IHC screenings of cancer tissues using large collections of mouse polyclonal and monoclonal antibodies towards secreted or membrane-associated poorly known human proteins [24]. An IHC screening of Tissue microarrays (TMAs) carrying clinical samples from each of the five major human cancers (breast, colon, lung, ovary and prostate, duplicated cancer and matched normal samples from 5 patients per each cancer) led to the identification of FAT1 as novel CRC associated protein.

**Fig. 2:**
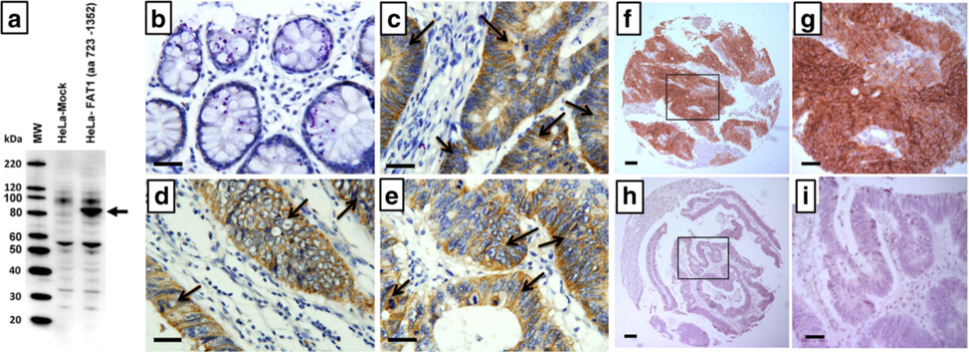
**a** Western blot analysis of HeLa cells over-expressing FAT1 with mAb198.3. HeLa cells were transiently transfected with pcDNA3-FAT1 plasmid and the empty vector (Mock). At 48 h post transfection HeLa cell total lysates (25 micrograms/lane, corresponding to approximately 105 cells) were loaded onto SDS-PAGE, transferred to nitrocellulose membranes and probed with mAb198.3. Molecular weight standards (MW) are reported on the left side. Arrow marks the expected protein size as determined from protein sequence. FAT1 detection in colon adenocarcinoma tissues by IHC. In contrast to normal colon tissues **b**, expression of FAT1 was significantly upregulated in colon adenocarcinoma tissues **c-e**. Bar, 50 μm. FAT1 detection in colon adenocarcinoma tissues by IHC. In contrast to non-adsorbed mAb198.3 **f-g**, the colon cancer tissue recognition was abolished by adsorbing the antibody on the FAT1 recombinant domain, further demonstrating the specificity of the antibody recognition **h-i**

In particular a murine mAb against a rFAT1 domain, generated in our laboratories, named as mAb198.3, emerged from the screening of hybridoma clones secreting mAbs against human FAT1 region from amino acid 723 to amino acid 1352 (domain 723–1352). The isotype of mAb198.3 was IgG1 (κ). The hybridoma was selected by ELISA screening on the rFAT1 protein. The mAb was produced as ascites and purified using affinity purification by ProteinG-Sepharose CL-4B [28]. MAb198.3 specificity was further confirmed by analyzing its ability to recognize FAT1 domain 723–1352 expressed in HeLa cells transfected with FAT1-encoding plasmid (Fig. 2a). MAb198.3 could be applied in IHC, confocal microscopy and flow cytometry on FAT1-transfected cells (data not shown).

### Expression profiles of FAT1 human colon carcinoma tissues and normal colon tissues detected by mAb198.3

The ability of the mAb198.3 to recognize CRC in IHC was demonstrated by Externautics (Grifantini et al., submitted manuscript). In this study, the expression of FAT1 was further verified in a new cohort of 43 patients affected by colon. In this experiment, all of the negative controls showed no specific positive reaction. High level expression of FAT1 was detected in 34 cases (79 %) of colon cancer tissues while there was no FAT1 expression in normal colon tissues (Fig. 2b). As shown in Fig. 2c-e, specifically recognized CRC showing a clear membranous staining that extended in part to the cell cytoplasm, with negligible staining in corresponding normal samples.

The specificity of the FAT1 recognition in colon cancer was further confirmed by pre-adsorbing mAb198.3 on nitrocellulose membrane coated with rFAT1 Domain and re-testing the antibody on colon cancer tissues. After adsorption, the antibody totally lost the ability to recognize FAT1 in Western blot analysis of total extracts of FAT1-transfected cells (not shown). Furthermore, mAb198.3 adsorption dramatically abolished the antibody reactivity on colon cancer, thus confirming the binding specificity (Fig. 2f-i).

### Reaction of mAb198.3 with native FAT1

**Fig. 3:**
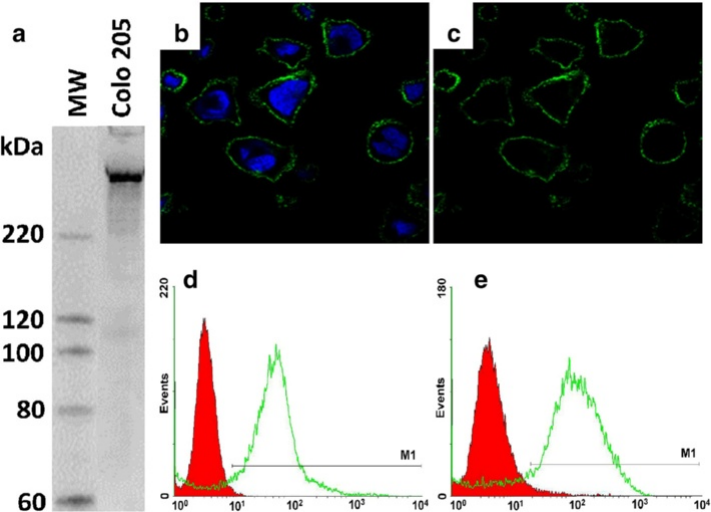
Expression analysis of FAT1 in Colo205 cells. **a** Western blot analysis of total Colo205 extracts. Colo205 were lysed, separated on SDS-PAGE (25 micrograms/lane, corresponding to approximately 10^5^ cells), transferred to nitrocellulose membranes and incubated with mAb198.3. Molecular weight standards (MW) are reported on the left side. **b-c** Confocal microscopy analysis: Cells were grown on glass coverslips, fixed and and incubated with the mAb198.3. Cells were stained with Alexafluor 488-labeled goat anti-mouse antibodies to detect FAT1 (green) and DAPI to visualize nuclei (blue). Flow cytometry analysis showing that both free mAb198.3_Cy5 **d** and AuCOOH(Cy5)_ mAb198.3 could bind Colo205 cells while the MFI of AuCOOH(Cy5)_ mAb198.3 is much higher than that of free mAb198.3_Cy5 **e**

In previous studies we found that FAT1 is expressed and surface exposed in a panel of colon cancer cell lines (Grifantini et al., submitted manuscript). Among the FAT1-positive cell lines, the colon cell line Colo205 was selected as model in the present study. Western blot analysis demonstrated the presence of a high molecular weight band of expected size (Fig. 3a). Confocal microscopy showed the FAT1 distribution at the plasma membrane of these cells (Fig. 3b-c). In addition, flow cytometry analysis demonstrated both free mAb198.3_Cy5 and AuCOOH(Cy5)_ mAb198.3 could bind to native FAT1 molecules on the surface of Colo205 cells (Fig. 3d-e). These results indicated that mAb198.3 could be used as potential target-specific delivery agents *in vivo*. Moreover, the mean fluorescence intensity (MFI) of AuCOOH(Cy5)_ mAb198.3 is much higher than that of free mAb198.3_Cy5 (Fig. 3e). This phenomenon was probably due to that Cy5 molecules loaded on Au nanoparticles were much more than that on mAb198.3.

### Characterization of Au nanoparticles

The DLS size distribution profile of the AuCOOH, AuCOOH(Cy5)_ mAb198.3, AuCOOH(Cy5)_ isotype shows that the modified gold nanoparticles are well dispersed in water, PBS buffer and cell culture medium with 10 % FBS. Additional file 1: Figure S1A summarizes the average particle size of AuCOOH, AuCOOH(Cy5)_ mAb198.3, AuCOOH(Cy5)_ isotype. The average particle size is about 12–24 nm with a narrow size distribution. And the zeta potential was also determined and shown as −342.78 mV for AuCOOH(Cy5)_ mAb198.3 and −692.28 mV for AuCOOH(Cy5)_ isotype in Additional file 1: Figure S1B. MAb usually increased surface charge when conjugated to nanoparticles [32, 33] and this generally results in increased tissue retention and increased blood clearance [34]. By TEM measurement, the size of Au nanoparticles with spherical morphology is about 10–20 nm (Additional file 1: Figure S1C).

**Fig. 4:**
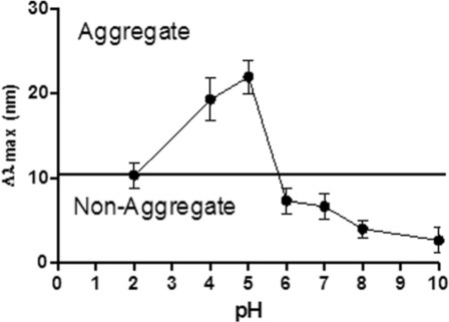
UV–vis spectra of AuCOOH_198.3 NPs at different pH values. A red-shift (Δλmax > 10 nm) is observed as the pH is decreased from 6 to 5, suggesting aggregation

**Fig. 5:**
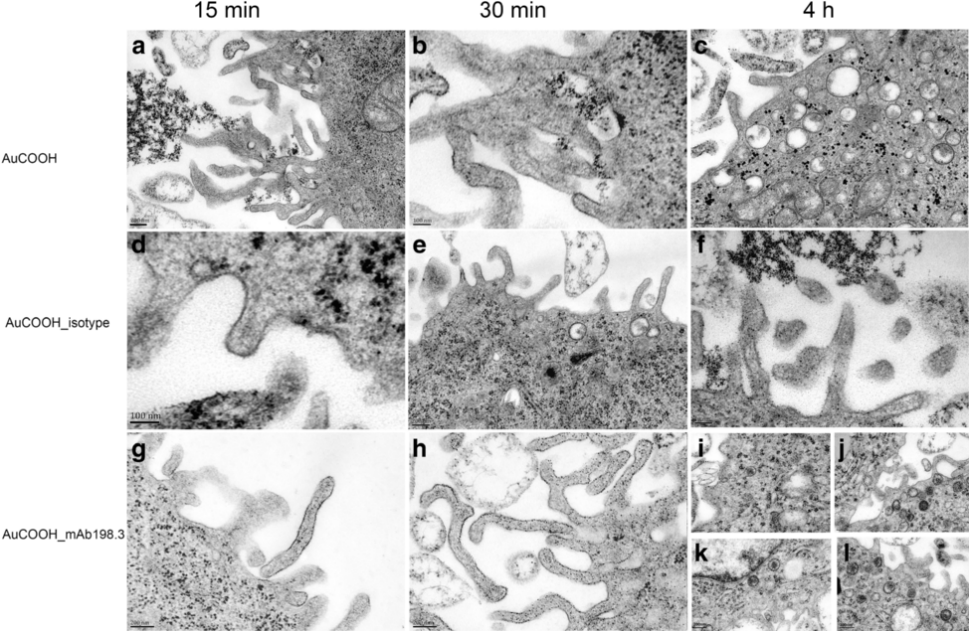
Transmission electron microscopy images of Au nanoparticles intracellular uptake by Colo 205 cells. After incubation with AuCOOH(**a**,15 min; **b**,30 min; and **c**,4 h), AuCOOH(Cy5)_ isotype (negative control) (**d**,15 min; **e**,30 min; and **f**,4 h) and AuCOOH(Cy5)_ mAb198.3 (**g**,15 min; **h**,30 min; and **i-l**,4 h), cells were collected and prepared for TEM analysis

Stability of AuCOOH_198.3 NPs in an acidic environment was investigated by incubating samples at varying pH values for 4 h. A red shift in maximal absorbance wavelength of the ultraviolet–visible (UV–vis) spectra in comparison to naked AuCOOH NPs (Δλmax > 10 nm) [35] was regarded as evidence of aggregation. Figure 4 shows that AuCOOH_198.3 NPs were found to aggregate below p H 5.8. This was consistent with the visible change in Fig. 5i-l, nanoparticles got aggregated inside endo-lysosomes (pH < 5.8) and became scattered in cytoplasm after endo-lysosome escape.

Moreover, stability of AuCOOH_mAb198.3 NP in PBS buffer, cell culture media and media with 10 % serum was also investigated by the same method above. No red-shift (Δλmax > 10 nm) is observed in three testing groups, suggesting no aggregation appears when AuCOOH_198.3 NPs dispersed in these three media (Additional file 1: Figure S2). All of the data are reported as the means ± S.D. Comparisons were carried out with a one-way analysis of variance (ANOVA) using GraphPad Prism 5.01 software. No significance was found as P >0.05.

### Cytotoxicity of Au nanoparticles

In this experiment, the toxicity of AuCOOH was studied to confirm biocompatibility and evaluate the safety. We quantified the toxicity of Au compounds in Colo205 cell line by determining the IC_50_ values (Additional file 1: Figure S3). Cytotoxic effects were not observed in Colo 205 cells treated with AuCOOH alone at the high concentration of 5 μM. The lack of significant growth inhibition by the free AuCOOH NPs indicated that this negative Au nanoparticles has well biocompatibility.

### Intracellular uptake of Au nanoparticles

Our previous studies showed that mAb198.3 was efficiently internalized in the Colo205 cell line when used as naked antibody (Grifantini et al., submitted manuscript). Next, we wished to confirm that mAb198.3 was able to drive the intracellular delivery of AuCOOH nanoparticles in cancer cell lines. To this aim, we have performed the intracellular uptake of AuCOOH_mAb198.3 in Colo 205 cells at different culture time point by cell TEM, as compared to AuCOOH, AuCOOH_ isotype as controls. It could be seen obviously that in AuCOOH_mAb198.3 group (Fig. 5g-l), the intracellular gold amount was significantly increased as culture time went by AuCOOH_mAb198.3 began to combine with the membrane microvilli in the first 15 min (Fig. 5g) and followed by free diffusion into the cells (Fig. 5h). In Fig. 5i-l, it could be observed very clearly that Au nanoparticles can go through the cell membrane into the cytoplasm and move towards cell nucleus (Fig. 5k). The procedure of Au nanoparticles passing the cell membrane through mAb198.3 mediated endocytosis process was clearly observed in Fig. 5i. Part of the plasma membrane was depressed to form small fossa, and fossa further separated from the plasma membrane to form coated vesicle. During this process, AuCOOH_mAb198.3 was brought into the cells. Plenty of Au nanoparticles was observed in endocytic vesicles near the cell membrane and in the cytoplasm (Fig. 5j and l), one step further confirming the active targeting effect of mAb198.3. In contrast, during the culture process within 4 h, AuCOOH(Cy5)_ isotype (Fig. 5d-f) and bare AuCOOH (Fig. 5a-c) showed very low uptake. Au nanoparticles were barely observed inside the cell and no particles were found in the endocytic vesicles (Fig. 5c). Glycogen granule (Gl), scattered in the cytoplasm, has similar morphological characteristics with Au nanoparticles except for larger size about 40 nm [36] in diameter. It should be strictly distinguished from Au nanoparticles. Gl can be easily identified after staining with lead, for instance. Their diameter is slightly larger than that of the nearby ribosomes (Ri) and averages 35 nm. The Gl usually occur in clusters and stain intensely. Overall, our results indicate that the conjugation of mAb198.3 on Au nanoparticles might be again an effective delivery vehicle for negatively charged gold nanoparticles and its increased intracellular transport.

**Fig. 6:**
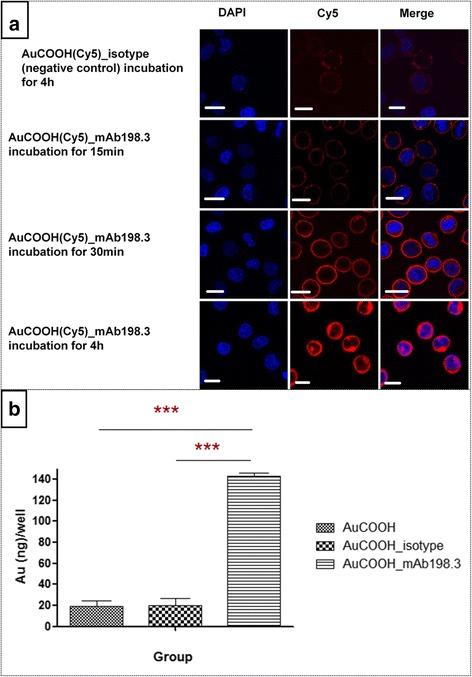
**a** Confocal images of Colo 205 cells incubated with AuCOOH(Cy5)_ isotype (negative control) and AuCOOH(Cy5)_ mAb198.3 and nucleus stained with DAPI. Incubated time: 15 min, 30,min and 4 h. (Blue fluorescence is associated with DAPI, and red fluorescence is associated with Cy5). Scale bar at 20 μm. **b** Quantitative ICP-MS analysis of the number of AuCOOH, AuCOOH_isotype and AuCOOH_mAb198.3 in colo-205 cells. All of the data are reported as the means ± S.D. Comparisons were performed with a one-way analysis of variance (ANOVA) followed by Bonferroni’s multiple comparison tests using GraphPad Prism 5.01 software. Significance was defined as P < 0.0001 (***)

Confocal microscopy was also used to monitor the cellular uptake process. In Fig. 6a, it was clear that mAb198.3 modified Au nanoparticles could attach to the cell membrane in very short time (about 15 min) through antigen antibody combination. After 30 min incubation, AuCOOH(Cy5)_mAb198.3 was gradually delivered into the cells. Cell membrane exhibited enhanced fluorescence intensity and fluorescence became emerged in cytoplasm. After 4 h incubation, almost all AuCOOH(Cy5)_mAb198.3 have been uptaken into or surrounding the cytoplasm and nucleus so that the cytoplasm presented bright red while the nucleus turned purple (Mixed red and blue made the color changed to purple). However, in the negative control group, AuCOOH(Cy5)_ isotype rarely entered the cells after 4 h incubation. The uptake efficiency increased greatly with the modification of mAb198.3 onto the Au nanoparticles surface. There could be two reasons for this phenomenon. On one hand, considering the biological factors, we assume AuCOOH(Cy5)_ mAb198.3 might enter cells via receptor-mediated endocytosis more efficiently than the AuCOOH(Cy5)_ isotype. Besides, some non-biological factors are also crucial and cannot be ignored such as particle size. Virtually, some larger and heavier particles such as AuCOOH(Cy5)_ mAb198.3 with average diameter of 24 nm can sediment more quickly than those smaller ones (AuCOOH(Cy5)_ isotype with average diameter of 12 nm) and therefore causing different dose of nanoparticles on the cell surface, thus dramatically influencing the uptake extent.

Quantitative analysis of the number of AuCOOH, AuCOOH_isotype and AuCOOH_mAb198.3 in colo-205 cells was also determined by ICP-MS. In case of AuCOOH_mAb198.3 (Fig. 6b), the intracellular gold amount was significantly increased in colo-205 cells compared to AuCOOH which showed very low uptake. Therefore, attaching of mAb198.3 on AuCOOH surface increased its intracellular transport. A similar uptake profile was observed for AuCOOH_isotype and AuCOOH_mAb198.3 in colo-205 cells, confirming that the presence of the isotype does not improve the uptake behavior in colo-205 cells due to a lack of internalization function. These results indicate that mAb198.3 can be an effective delivery vehicle for negatively charged gold nanoparticles.

Therefore, from the above results of intracellular uptake in vitro, AuCOOH(Cy5)_ mAb198.3 are nontoxic to normal cells and could be potential target-specific delivery agents.

### In vivo bio-imaging study

**Fig. 7:**
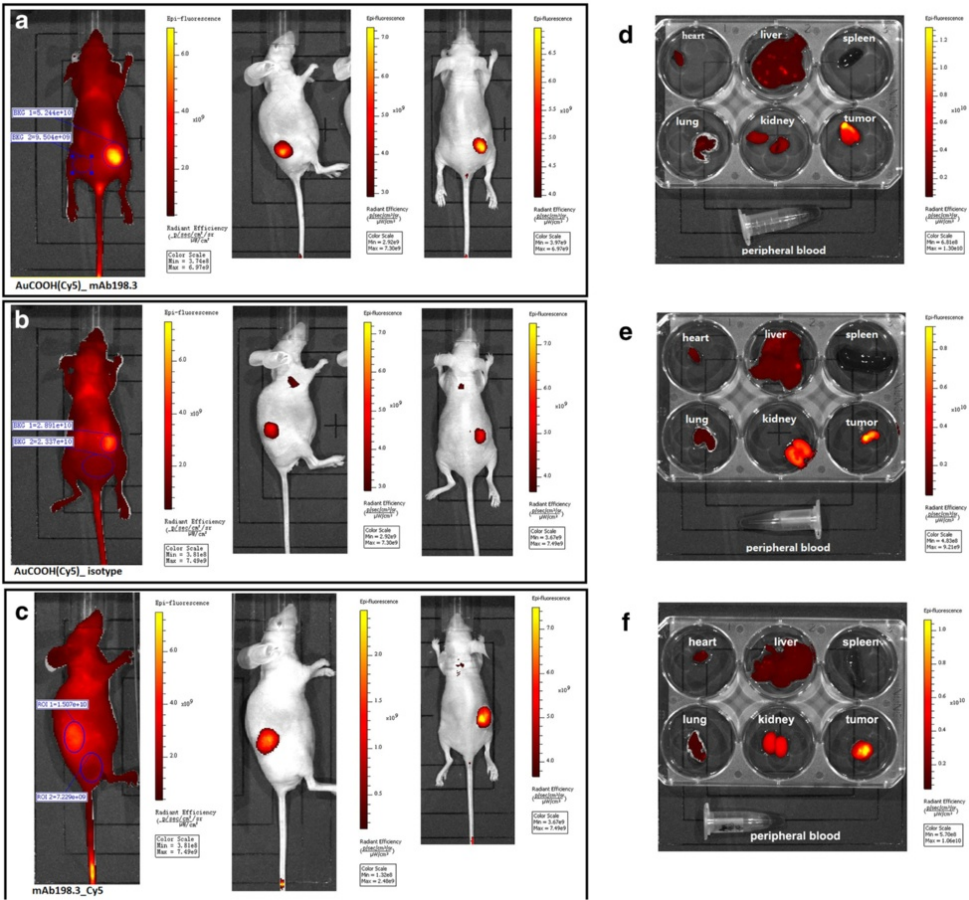
In vivo imaging of Colo 205 tumor bearing mice. Fluorescent signal captured by IVIS Lumina Imaging System in tumor bearing mice after injection with AuCOOH(Cy5)_ mAb198.3 (**a**), AuCOOH(Cy5)_ isotype (**b**), and mAb198.3_Cy5 (**c**) for 24 h. Luminescent image of resected organs from Colo 205 tumor-bearing mouse injected with AuCOOH(Cy5)_ mAb198.3 (**d**), AuCOOH(Cy5)_ isotype (**e**), and mAb198.3_Cy5 (**f**) for 24 h

In order to demonstrate the targeting efficiency of AuCOOH(Cy5)_ mAb198.3 in vivo, bio-imaging study was conducted using in vivo fluorescence imaging system. Fluorescent images of treated mice were recorded at 24 h post injection using an IVIS® Spectrum Imaging System. As shown in Fig. 7a, intense fluorescence from the tumor site can be clearly distinguished, due to mAb198.3 guiding effect and EPR effect. On the contrary, weak fluorescence from the AuCOOH(Cy5)_ isotype treated cells can be detected 24 h post injection (Fig. 7b). Still, some of AuCOOH(Cy5)_ isotype could targeting delivery to tumor site due to EPR effect. Quantitative evaluation of the integrated fluorescence intensity in tumor site (ROI, marked with blue circles of the same size in Fig. 7) upon injection of AuCOOH(Cy5)_ mAb198.3, AuCOOH(Cy5)_ isotype, mAb198.3_Cy5 and isotype_Cy5 were conducted using IVIS® Spectrum imaging software after substraction of auto fluorescence (Fig. 7). The fluorescence intensity (4.294 × 10^10^) of AuCOOH(Cy5)_ mAb198.3 treated group is ~9-fold higher than that of AuCOOH(Cy5)_ isotype treated ones (5.54 × 10^9^) and ~5-fold higher than that of mAb198.3_Cy5 group (7.84 × 10^9^) (Fig. 7c) upon 24 h post injection. In order to further substantiate tumor-specific targeting from passive accumulation of AuCOOH and provide sufficient evidence for receptor-specific targeting of AuCOOH_mAb198.3, in-vivo quantification of NPs was determined by ICP-MS analysis in three treatment groups (AuCOOH, AuCOOH_isotype and AuCOOH_mAb198.3). Additional file 1: Figure S4 shows that, only about 20 % of AuCOOH accumulated at the tumor site due to the EPR effect, while nearly 90 % of AuCOOH_mAb198.3 was found in the tumor, providing sufficient evidence for receptor-specific targeting by mAb198.3. To investigate the biodistribution of AuCOOH(Cy5)_ mAb198.3, the mice were sacrificed and the major organs and tumors were collected for imaging after intravenous injection of AuCOOH(Cy5)_ mAb198.3, AuCOOH(Cy5)_ isotype and mAb198.3_Cy5 for 24 h. Figure 7d-f show luminescent images of heart, liver, spleen, lung, and kidney. As observed from the color of Cy5, the Au nanoparticles are mainly accumulated in the tumor, with some accumulation in the liver and kidney, while there was no accumulation in the spleen and peripheral blood. The results revealed that weak fluorescence was detected in the mouse tumors after 24 h of treatment with AuCOOH(Cy5)_ isotyIntracellular negativepe and relative stronger fluorescence mainly accumulated in the kidney (Fig. 7e). Xenograft mice were likewise treated with mAb198.3_Cy5 (Fig. 7f). Conjugation of Cy5 to mAb198.3 resulted in about a 15.5-fold increase in the delivery of Cy5 to tumor tissue compared to the delivery of AuCOOH(Cy5)_ isotype (ROI = 51.41). MAb198.3_Cy5 treatment resulted in a high level of fluorescence intensity (ROI = 799.7) in the tumor and liver. However, treatment with AuCOOH(Cy5)_ mAb198.3 resulted in highest level of fluorescence intensity (ROI = 1465) in the tumor (about 28.4-fold compared with AuCOOH(Cy5)_ isotype treated group) and a negligible intensity in the spleen and normal liver tissue(Fig. 7d). In the AuCOOH(Cy5)_ isotype group we observed a strong fluorescence intensity which accumulated in liver and kidney while a weak fluorescence intensity accumulated in the tumor. Overall, our results suggest that mAb_198.3 has a strong targeting efficiency. It is worth mentioning that in the AuCOOH(Cy5)_ mAb198.3 treated group, an enhanced fluorescence could be detected in liver metastases tissue. It also showed successful tumor delivery of Au nanoparticles.

## Conclusion

Herein, we demonstrated the use of a novel tumor targeting antibody directed against colon expressed FAT1 (clone mAb198.3) used for intracellular negative gold nanoparticles delivery. The size of mAb198.3 coated Au nanoparticles with spherical morphology is about 20 nm according to the DLS results with negative zeta potential. Our data further reveal that the system is stable both in PBS and medium with serum. According to in vitro cellular intracellular uptake and in vivo bio-imaging studies, the intracellular uptake of negatively charged AuCOOH_mAB198.3 particles is enhanced to a greater extent. Thus, this system accumulation holds significant potential to improve the treatment of cancer.

## Additional file

Additional file 1:
**Supplementary figures.** (DOC 2242 kb)

## Abbreviations

TEM: Transmission electron microscopy
DLS: Dynamic light scattering
ICP-MS: Inductively coupled plasma mass spectrometry
CRC: Colorectal cancer
IHC: Immunohistochemistry
DCM: Dichloromethane
AuCOOH: COOH-PEG-SH capped gold nanoparticles
rFAT1: Recombinant FAT1
TCEP: Tris(2-carboxyethyl)-phosphine hydrochloride
EDC: 1-(3-dimethylaminopropyl)-3-ethylcarbodiimide hydrochloride
DMSO: Dimethyl sulfoxide
PBS: Phosphate buffered saline
FPLC: Fast protein liquid chromatography
MTT: 3-[4,5-dimethylthiazol-2-yl]-2,5-diphenyltetrazolium bromide
Gl: Glycogen granules
