# Supplementary material for: Negatively charged AuNP modified with monoclonal antibody against novel tumor antigen FAT1 for tumor targeting
Source: J Exp Clin Cancer Res. 2015 Sep 15;34(1):103. doi: 10.1186/s13046-015-0214-x (PMC4570718; doi:10.1186/s13046-015-0214-x)
Supplement: Additional file 1: — Supplementary figures. (DOC 2242 kb) [file 13046_2015_214_MOESM1_ESM.doc]

**Supplementary Figures**


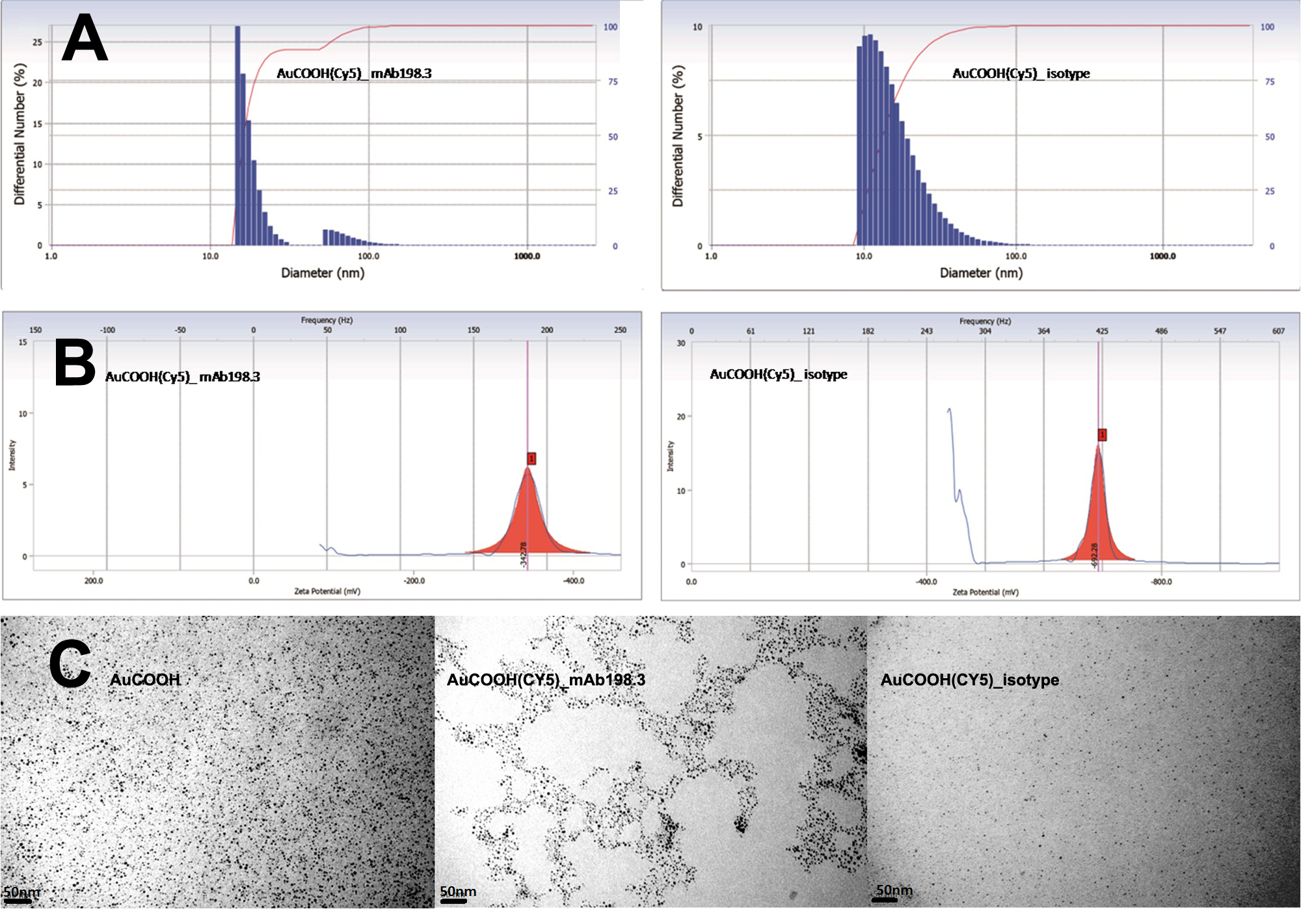


Figure S1. DLS size distribution(A), zeta potential(B) profile and TEM photograph(C) of modified AuCOOH

Figure S2. UV-Vis spectra of AuCOOH_198.3 NPs in different media. No red-shift (Δλmax>10 nm) is observed in three testing group, suggesting no aggregation appears when AuCOOH_198.3 NPs dispersed in these three media. All of the data are reported as the means ± S.D. Comparisons were performed with a one-way analysis of variance (ANOVA) using GraphPad Prism 5.01 software. No significance was defined as P >0.05.


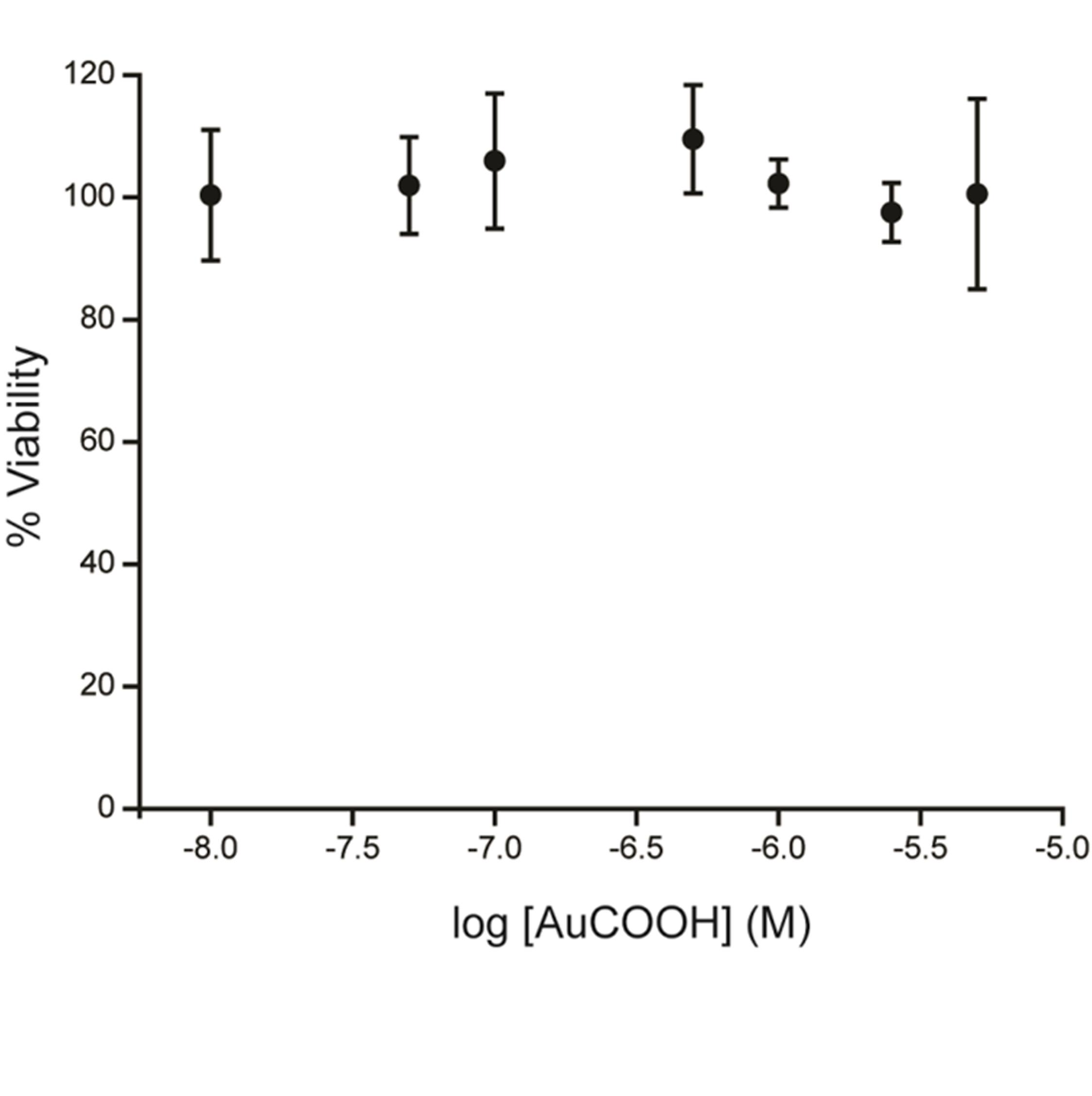


Figure S3. Cytotoxicity of of AuCOOH in Colo 205 cell line

In order to substantiate tumor-specific targeting from passive accumulation of AuCOOH and provide sufficient evidence for receptor-specific targeting of AuCOOH_mAb198.3, in-vivo quantification of NPs was determined by ICP-MS analysis in three treatment groups (AuCOOH, AuCOOH_isotype and AuCOOH_mAb198.3) in tumors 24 h after injection to allow for sufficient time for the onset of EPR effect. After 24 h, cancerous mice will be sacrificed for the collection of the tumors. Weigh known amount of tumor tissue and digest it with nitric acid: perchloric acid (3:1) (30 ml: 10 ml) with heating and stirring at 200°C till the volume reaches 5 ml then complete with distilled water till 10 or 15 ml. Measure the gold with ICP-MS.

Figure S4. Quantitative ICP-MS analysis of the number of AuCOOH, AuCOOH_isotype and AuCOOH_mAb198.3 in each treated tumor. All of the data are reported as the means ± S.D. Comparisons were performed with a one-way analysis of variance (ANOVA) followed by Bonferroni’s multiple comparison tests using GraphPad Prism 5.01 software. Significance was defined as P < 0.01 (**).
